# Navigating change – managers’ experience of implementation processes in disability health care: a qualitative study

**DOI:** 10.1186/s12913-021-06570-6

**Published:** 2021-06-10

**Authors:** Anette Granberg, Marie Matérne, Lars-Olov Lundqvist, Anna Duberg

**Affiliations:** 1 University Health Care Research Center, Faculty of Medicine and Health, Orebro University, Orebro, Sweden; 2 The Swedish Institute for Disability Research, Orebro University, Orebro, Sweden

**Keywords:** Implementation processes, management, leadership, disability, change

## Abstract

**Background:**

Effective implementation processes play a central role in health care organizations and affect the care of patients. Managers are pivotal in facilitating the use of new practices, but their experience and how it affects the implementation outcome are still largely unknown. In the field of disability health care in particular, managers experiences have scarcely been investigated. Therefore, the aim of this study is to explore managers’ experiences of the implementation process when transferring new practices into disability health care settings.

**Methods:**

Semi-structured individual telephone interviews were conducted with managers at disability health care organizations in four administrative regions in central Sweden. A total of 23 managers with formal managerial responsibility from both public and private health care were strategically selected to be interviewed. The interviews were analysed using reflexive thematic analysis with an inductive approach.

**Results:**

The analysis resulted in two themes about factors influencing the implementation process: firstly, *Contextual factors set the agenda for what can be achieved*, which highlighted aspects that hinder or enable the implementation process, such as internal and external conditions, the workplace culture, the employees and managers’ attitudes and openness to change: secondly, *Leadership in the winds of change*, which described the challenges of balancing managerial tasks with leading the change, and the importance of a leadership that involves the participation of the employees.

**Conclusions:**

This study explored how and to what extent managers address and manage the implementation process and the many associated challenges. The findings highlight the importance of leadership support and organizational structure in order to transfer new practices into the work setting, and to encourage an organizational culture for leading change that promotes positive outcomes. We suggest that identifying strategies by focusing on contextual factors and on aspects of leadership will facilitate implementation processes.

**Trial registration:**

The SWAN (Structured Water Dance Intervention) study was retrospectively registered on April 9, 2019 and is available online at ClinicalTrials.gov (ID: NCT03908801).

**Supplementary Information:**

The online version contains supplementary material available at 10.1186/s12913-021-06570-6.

## Background

The relevance of leadership in implementation research has been increasingly acknowledged in recent years [1, 2]. Although managers play an important role in facilitating the use of new practices by making a process easy or easier, or by promoting and helping the process forward [3], their experience of the implementation process in health care, and how it affects the implementation outcome, are still largely unknown [4–7]. The term *implementation process* refers to using or integrating new practices within a particular setting [8].

For the desired use of new practices in health care routines to be achieved, several studies point out the importance of increased knowledge about what factors influence implementation processes from the managers’ perspective [1, 9–11]. It is also considered important to understand how managers interpret and construct an implementation project in the light of those factors [6].

The difficulty of introducing new practices has led to an increased focus on research to identify barriers and facilitators for implementation and to find strategies to foster more evidence-based practice [12–14]. In health care, it is important to acknowledge social processes for implementation [14, 15]. According to Neal et al. [16], a social process requires the implementers to communicate with and receive social support from multiple actors including other implementers, researchers, support staff, and senior management [16]. These social processes exist in a context, that is, a set of characteristics and circumstances that form active and unique aspects of the implementation [17]. The context is an important component [18–21] because it influences, facilitates and constrains the implementation process [17]. A review of determinant frameworks [11] showed that the most widely addressed context dimensions for implementation were organizational support, financial resources, social relations and leadership. However, it is not clear how and to what extent the context influences the implementation process [20].

A number of theories [22–24], models and frameworks [3, 25–28] have been developed to guide and plan the implementation process to reach a desired outcome [29, 30]. The Integrated Promoting Action on Research Implementation in Health Services (i-PARIHS) framework [3] has been widely used as an organizational and conceptual framework to help explain and predict the success of an implementation process [3]. The implementation process can be a major challenge in health care settings, and empirical studies suggest that the success rate of implementations of evidence-based practices in health care is less than 50 % [31, 32]. Consequently, there is a certain risk that patients are missing effective treatments or receiving unnecessary treatment [8, 33, 34]. This is critical, not only for patients, who thereby fail to receive the best available treatment, but also for healthcare organizations and society, who miss out on the potential financial benefits and returns on investment [35].

In the Swedish health and welfare services, some areas have come a long way in implementing new practices, for example surgical routines in hospitals as well as some parts of individual and family care in the social services [36]. The field of disability has not developed as far [37]. The reason for this is not known, but extensive and varied needs among patients are mentioned as one possible reason, as well as the low education level among the employees and a lack of a tradition of implementing new practices [37]. The multifaceted needs of patients with disability and the diverse practice settings create a fragmented system that is difficult to navigate [38].

To be able to identify the roles of the professionals and their influences on the implementation processes in detail, more in-depth studies are required [37]. Therefore, the aim of this study was to explore the experiences of disability health care managers when leading implementation processes. By exploring this issue from the managers’ perspective, we strive to gain a deeper understanding of their views on what factors influence implementation processes.

## Method

### Design and participants

We used a semi-structured individual interview design with managers at private companies that provide personal assistance and with managers at habilitation centres in four regions in central Sweden. We conducted telephone interviews with managers in both public and private disability health care organizations who had formal managerial responsibility and were responsible for leading implementation processes in their organizations. We gathered a strategic sample by recruiting the managers from disability health care organizations participating in a structured water dance intervention study (SWAN) for adults with profound intellectual and multiple disabilities. The intervention is described elsewhere [39, 40]. The managers were recruited specifically to shed light on the phenomenon being studied. Its composition reflected our interest on what aspects influenced implementation processes from the managers’ experience. The size of the sample was determined by the availability of respondents and resources to complete the study.

In total, 34 managers were identified. The first author (AG) contacted all managers to invite them to participate in an interview to study their experience of implementation processes. Eleven managers declined participation, did not respond to the invitation or indicated that they were on sick leave and therefore did not want to participate (women n = 7, men n = 4). The remaining 23 managers agreed to participate. The heterogeneity of the participating managers was reflected in the variety of public and private health care organizations they represented, as well as sector variations in the four regions, see Table 1.

**Table 1:** Participant characteristics

	Private sector *n* = 10	Municipal sector *n* = 7	Regional sector *n* = 6
Sex Women Men	9 1	7 0	5 1
Years as manager, mean (range)	12.4 (1–25)	13.4 (0–30)	16.3 (2–30)
Years of post-secondary education or vocational training mean (range)	2.3 (0–3.5)	3.4 (3–5)	3.2 (3–3.5)

### Data collection

The integrated PARIHS (i-PARIHS) framework was used as an inspiration for the development of the interview guide [3]. The framework consists of a multi-dimensional structure, reflecting the complex nature of implementation and the central importance of context [3]. The interview guide consisted of questions regarding different areas of the implementation process, namely, innovation, recipients, context and facilitation. Before conducting the interviews, the interview guide was tested on three managers in other business areas but within the same organization as the participating managers. The participants were informed about the purpose of the study, assured of confidentiality and reminded that they were free to terminate their participation in the study at any time. All participants signed a consent form. The interview questions were sent out to the participants one week before the individual semi-structured telephone interviews.

 Depending on how the participants responded, follow-up questions were asked to find out more about the managers’ experience and to elicit detailed descriptions about the implementation process from their point of view. The interviews were recorded, and the length of the interviews varied between 50 and 75 min. The first author (AG) performed and transcribed all interviews verbatim. The transcribed documents were stored in data files only accessible by the research team.

### Data analysis

The interviews were analysed using reflexive thematic analysis (TA) as described by Braun and Clarke [41]. Reflexive TA was chosen because the method can capture rich material and provide a good structure for the analysis. We used an inductive approach to generate rich descriptions of the managers’ experience of the implementation process. First, we *familiarized ourselves with the data*. This step was achieved by reading and rereading each transcript and making notes in the margin. All of the authors (AG, MM, LOL, AD) were engaged in this part of the analysis. In the second step, the first author (AG) *generated initial codes* from the material, and then AG and AD grouped them together. The NVivo 12 qualitative data-analysis software was used [42]. The first author (AG) made the first draft in the third step, *searching for themes*, and suggested relevant themes from the initial codes. The subsequent steps included *reviewing, defining and naming themes*, for which all the authors were engaged. Different possible interpretations were discussed among the authors, which generated additional understanding of the material. The final results emerged through consensus and resulted in five sub-themes and two themes. The SRQR standards assisted author (AG) during manuscript preparation (see Additional file 1).

## Results

**Fig. 1:**
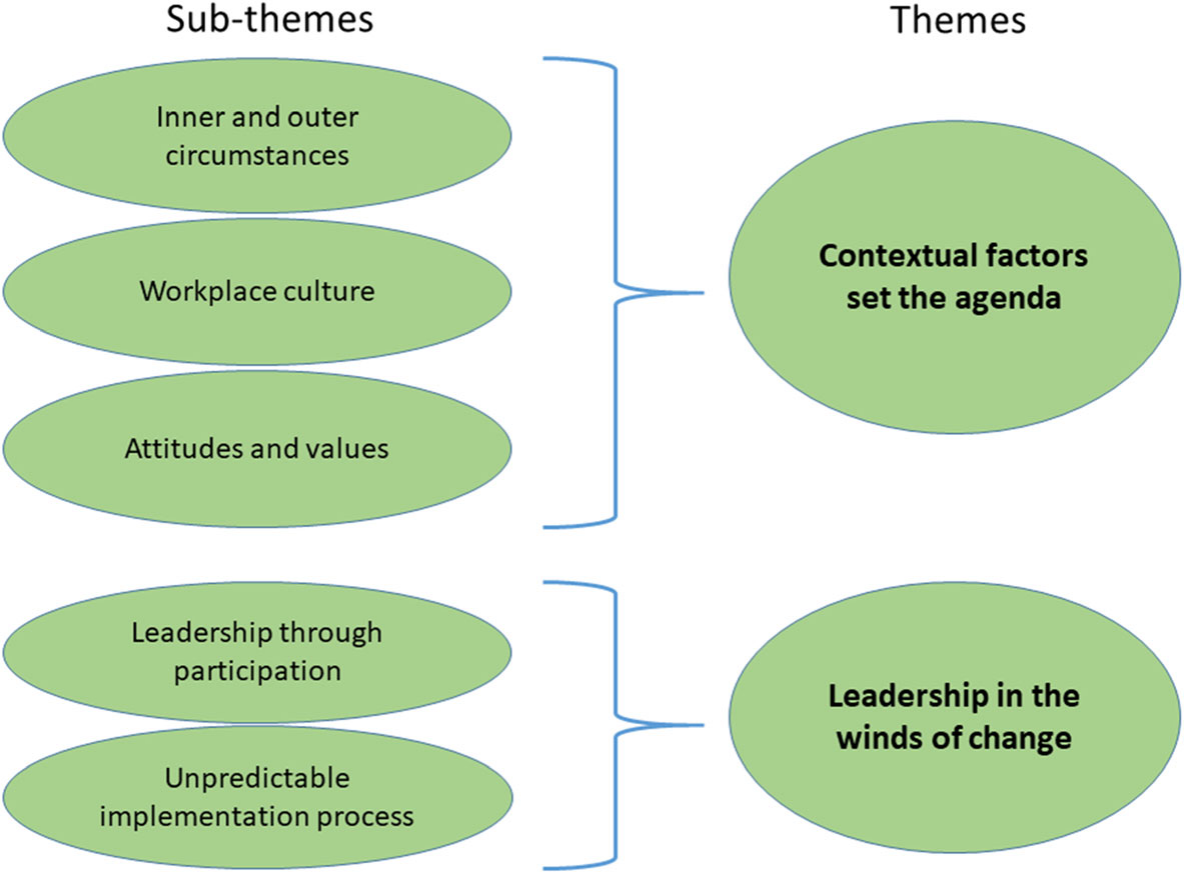
Final thematic map showing five sub-themes and two themes

The analysis identified two themes: *Contextual factors set the agenda for what can be achieved* and *Leadership in the winds of change*. The two themes encapsulate how the managers struggled to deal with various constraints they had to relate to, and simultaneously found time to perform the implementation processes. This struggle with sometimes conflicting demands led to difficulties balancing between managerial duties and leading change. Within these two themes, the managers described a number of factors that acted as barriers or enablers to the implementation process, which could be classified into the five sub-themes (Fig. 1). All themes and sub-themes are outlined below, with quotations from managers in the private sector (PS), municipal sector (MS) or regional sector (RS) to illustrate the findings.

### Theme – Contextual factors set the agenda for what can be achieved

#### Sub-theme – inner and outer circumstances

Disability organizations often require a team of professionals to ensure that patients receive comprehensive care. From the internal perspective, managers stressed the importance of having functional teams that collaborated on the care of the patients. Some managers expressed difficulties with team collaboration because some employees could not adapt to the change that the implementation entailed. Other managers described a lack of collaboration between professionals at the workplace, other than collaboration concerning the patients. Some managers believed that the lack of collaboration was a result of poorly defined and unclear roles and responsibilities in the teams.

(…) we have one of those large woolly assignments that you can always get pretty well everything into (…) we tried to work just on this how can we do an investigation together, how can we collaborate and change, we don’t need to lock ourselves in the team we are employed in (…)

*(Interviewee 6/RS)*

From an external perspective, many managers described a lack of communication between government authorities such as the Swedish social insurance agency, municipality and regional administrations, which adversely affected the implementation process and sometimes created misunderstandings and mistrust among some managers. Sometimes the lack of communication resulted in a clear conflict of interest, possibly because of different agendas.

*“(…) and also differences between administrators, municipalities, offices, and how they relate to laws and guidelines obviously affects both our clients and our work. In what you think you should help with or what support they are entitled to and so on (…), of course it affects (…) people may be less inclined to dare to drive change when it is unsettled all around.” (Interviewee 12/PS)*.

All managers highlighted the importance of support from their organization in terms of financial and personnel resources to carry out the implementation process. Sometimes they felt that managers at higher levels in the organizations demanded other priorities and had different expectations, which made it difficult for the interviewed managers to prioritize their own work.

*“(…) it is a constant balance between how much can be set aside for change work when we still need to produce habilitation so to speak for the individuals, it is a problem, or it can be a problem.” (Interviewee 1/RS)*.

Another factor that affected the implementation process was political decisions. These decisions controlled and influenced the work in different ways, particularly by setting the financial budget and thus limiting the type of activities that could be carried out. Most managers addressed the issue of politicians’ supposed lack of knowledge and understanding of the consequences that their decisions had for the patients; they experienced this as a major problem to deal with.

*“(…) the politicians often want us to both save and invest at the same time, and sometimes, (…) it becomes challenging, (…). We try as far as possible so that the patients are not affected by the decisions.” (Interviewee 9/MS)*.

#### Sub-theme - workplace culture

Meeting the preconditions for creating a stimulating culture and having realistic expectations of what work can be performed were considered important among the managers. However, despite their efforts to create an environment optimized for change, they experienced that the employees were differently disposed to change. All managers stated that it was obvious that most employees were more motivated to change if it concerned the patients’ wellbeing; however, this could be challenging when other aspects of work needed to be changed and developed.

*“In the daily work with our clients they are very willing to change and flexible because it requires it (…) but when it comes to rules and routines around (…) for example food expenses and how we handle educational meals and things like that, then they are not as positively minded.” (Interviewee 21/MS)*.

The managers expressed the opinion that some employees were less inclined to implement changes if it involved administration duties, initiating new relationships or treating new patients instead of keeping their usual patients. Some managers described this workplace culture as the “the culture of satisfaction”, in which no change was needed.

*“(…) now I sound a little pessimistic, which I’m not really, but (…) there is a culture that says it is fine as it is, something like that. Yes, some satisfaction … culture of satisfaction I would say.” (Interviewee 6/RS)*.

Some managers wondered if the employees had fallen into a habit culture where they performed tasks automatically.

*“How much does the ingrained culture play a role in their daily work? Has it taken over, or are they still objective in their work, are they still themselves when working with the residents or have they fallen into something that people do automatically at that workplace (…)?” (Interviewee 2/MS).*

Managers thought that some employees felt threatened by any change and therefore preferred to rely on their own expertise, such as creating their own routines and culture. In some habilitation centres, the managers reported that the employees considered their workplace as unique and very special because they worked with patients with special needs.

*“(…) we work with people who have specific and special needs, so we can easily take an outsider position (…) no, this won’t work for us. So that we have had the opportunity to stand a little to one side (…), and I think we have worked really hard to get, like, habilitation is a part of health care.” (Interviewee 22/RS)*.

#### Sub-theme – attitudes and values

The managers described how their own and their employees’ attitudes and ability to change influenced the implementation processes. Some of the managers considered that the employees’ enthusiasm for participating in change depended on their age, education level and work experience. Most of the managers observed that younger people with higher education and experience from other workplaces were generally more inclined to change. The managers’ experience was that these employees were more committed, had more drive and had a greater ability to see opportunities instead of problems in change and therefore were more positive to the implementation process. The managers also perceived that employees who lacked understanding of the implementation, not being driven by implementation success, were not able to prioritize and take direction, which had a more negative effect on the implementation process.

*“I mean, you would like change to happen by someone else changing, just not me. The others can change but just not me.” (Interviewee 4/RS)*.

A few managers found it hard to work with change when employees sometimes “only saw to their own good”, and perceived any kind of suggested change as a direct criticism of their own previous work – ‘Isn’t our work good enough?’ These negative attitudes were considered a risk for negatively influencing other employees and teams. Some managers commented that this was characteristic of dysfunctional teams. However, few managers reflected upon their own role and responsibility for these employees’ lack of will to change and their own negative attitude towards change.

*“For me it is problematic that there are employees who find it hard to change and then I think that part of it, (…) that they have some kind of logic that what they have done before was not good enough (…).” (Interviewee 1/RS)*.

The managers expressed the opinion that the employees sometimes had a view of having their own special expertise with disabled people, who therefore needed them or were dependent on them. Consequently, any change that could be perceived as criticism of their abilities would threaten the employees’ expertise or perhaps their professional identity. Managers found it hard to implement change because sometimes the employees argued that it was unnecessary because they had tried this change before, or that there was nothing wrong with the way they currently did the work.

*“Instead of bringing new patients, new relationships (…), making new diagnoses, (…), because it requires more, it is comfortable to fill the day with something very familiar (…) I have problems with employees who (…) it is too much sympathy, and we can’t carry the entire population’s health on our shoulders (…).” (Interviewee 4/RS)*.

Some managers expressed that their organization met the preconditions for change. An important success factor was that the demands and expectations for employees’ knowledge and skills were high. The managers experienced this as problematic because the employees themselves felt that they had enough knowledge and competence to cope with the work and were less interested in their own professional development.

*“They think they have enough knowledge and training for their work, (…) I feel that they cannot say or see what they need to work on, or what they would like more of, but rather they feel quite accomplished in terms of competence.” (Interviewee 11/PS)*.

The managers speculated that the employees’ lack of motivation and ability was possibly related to top-down decisions and that the employees could not see the context or reason for implementing the change. Some managers described leading the implementation process as a balance between pushing forward and sometimes reversing the process, depending on the changing situation.

*“(…) I am very flexible and inclined to change, (…) but you must not be too naive in this, because you have to get people to go along with it, (…) as a leader, it’s not always easy. You have to reverse a little, pull a little on the handbrake, accelerate, reverse, accelerate.” (Interviewee 2/MS)*.

### Theme – leadership in the winds of change

#### Sub-theme – leadership through participation

The managers underlined the importance of a clear and empathetic leadership, and they believed that it was important to have a reflective approach on the issue of what characterizes good leadership. They also highlighted a number of challenges to leading the often very unpredictable implementation processes, which could be initiated based on unclear needs, with insufficient time and space to lead the change and a lack of follow-up; they often found it difficult to prioritize. Most of the managers highlighted the importance of involving and creating a context in which all employees participated throughout the implementation process.

*“Yes, it makes such a difference (…) when employees can be involved and know that they have been invited to some form of participation then most employees buy into the change.” (Interviewee 14/RS)*.

All managers expressed a clear idea of what was included in an implementation process. One aspect that managers highlighted as particularly important was communication of the content and objective of the implementation process to employees. To engage all employees in the implementation process, some managers stressed the importance of satisfying the employees’ various needs in working with change, such as having different approaches depending on the situation and adapting to the employees’ individual needs. Other managers explicitly highlighted the importance of employees understanding the change and the reasons behind it.

*“Yes, one really important factor is communication and time. In order for me to get a change process established it is not enough to write it in a business plan, to reach effectiveness we have to plan in order for the things that will actually carry out this change and to understand why we should do it (…).” (Interviewee 23/RS)*.

Informal leaders were regarded as key players early in the implementation process. Identifying and encouraging informal leaders was essential for the managers. Some managers had an explicit approach to handling negative informal leaders, by having individual conversations and follow-ups to improve the employees’ attitude to change. The positive informal leaders were typically highly engaged in the tasks and could help the implementation process forward. The managers’ explanation for this was often “because they got the job done”, and some positive informal leaders were able to convince potential opponents better than the managers could themselves. Some employees were more sceptical about their managers’ motivation for change than about the informal leaders’ motivation for change.

*“It is often those who give the change management a boost (…) you make sure to have them with you in the process at an early stage. Sometimes they can convince any opponents to join better than I can (…) because I am their manager they can have a sceptical attitude to me because I might have other motives for change, such as saving money.” (Interviewee 13/MS)*.

#### Sub-theme – unpredictable implementation process

Leading the implementation process was mostly experienced as challenging, and the managers’ narratives mentioned examples such as unclear needs, lack of follow-up or evaluation and the difficulty in assessing the rationality of the implementation work based on the prevailing circumstances. Most of the managers were struggling with difficulties communicating with the employees, trying to create an understanding of the purpose of the implementation process and the estimated benefits of the change. Some managers experienced this as exhausting and some of them expressed resignation.

*“(…) then we noticed that they still had not understood even though we had gone through it a lot. The problem I think is to make it work fully. There are always some individuals who do not fully understand even if we make every effort.” (Interviewee 8/PS)*.

The managers expressed some problems keeping up with the fast pace of change and some challenges adjusting to new knowledge. Some managers highlighted poor conditions for implementing new practice. For example, that change sometimes did not match the mission of the organization, was not based on needs of the patients, lacked evidence for the methods or did not fit the organization’s overall strategic plans. Some managers explicitly stated that the change should be based on patients’ needs and respect their right to equal care. Others highlighted evidence-based practice as the most important precondition because it creates motivation among employees. The use of evidence-based practice was significant for some workplaces with highly development-oriented work, but some managers found it hard to identify why some methods were much faster to implement than others.

*“Sometimes it is something that comes out of the blue and is introduced in no time at all, and for some reason it goes really easily sometimes. It can be a very narrow interest (…) sometimes you may not know so much about the evidence either and it can go madly fast at times.” (Interviewee 1/RS)*.

Managers experienced a lack of time for working with implementation processes. This was expressed as “too overwhelming to handle” because nothing else was removed from their ordinary workload and every new task added to their burden. Some managers found it difficult to keep control of what had been agreed upon and to continue working towards common goals.

*“You also have to evaluate what does not work that is not beneficial for the patients, and remove things that are not helping (…) We are quite good in health care at bringing in things but we are pretty bad at removing things.” (Interviewee 4/RS)*.

In general, the managers experienced the quality of follow-up and evaluation as substandard, which led to difficulties in knowing what actually worked or did not work in everyday situations. Some managers believed that it was important to acknowledge the complexity of the implementation process and to consider that even small and simple changes required time, attention and reflection.

*“(…) we visualize all changes big and small. I had problems justifying why we should do it. Now I understand that showing how much we have done (…) gives rise to a desire to change when one sees something is happening.” (Interviewee 14/RS)*.

Overall, there were no differences in the managers’ experiences of the implementation processes, despite the different experience time. The managers with the most experience seemed clearer in their descriptions of how they included staff in the change work. On the other hand, the managers who had less experience could relate to previous employments and thus provided a rich description of the topic being studied.

## Discussion

The aim of this study was to explore managers’ experiences in disability health care when leading implementation processes. The managers expressed a rich palette of experiences. The results reveal the challenges managers face related to how and to what extent they address and manage the implementation process and frequent challenges. The way the managers interpreted and made sense of the process was influenced by contextual factors inside and outside the organization, which set the agenda for what could be achieved. The managers perceived their leadership as situated in the winds of change in relation to these contextual factors, and they described the challenge of leading and handling unpredictable implementation processes.

An interesting finding was how the managers perceived and expressed their ordinary day-to-day managerial work as distinct from leading change and how difficult they found it to balance these two processes. Somehow, they saw managerial work and leading change as completely different and separate parts of their role. This may be understood as based on the conflict between the function of management and that of leadership. Similarities and differences between leadership and management are widely discussed in the literature [43–46], but there are few empirical studies that investigate the role and functions of leadership and management [47].

Our results showed that the implementation processes were hampered by various contextual factors, mostly situated in the local environment, such as employees’ resistance to change, opposition from informal leaders, weak collaboration networks or teams, lack of support from senior management and lack of organizational structure within the process. This finding confirms findings from other studies showing similar organizational barriers restricting the implementation process [18, 21, 25, 48].

Another barrier revealed by the interviews was the competing pressure of managerial work and leading change, as the prevailing circumstances changed due to the generally increasing pace of change in the health care sector. Since changes in health care occur so rapidly, they are less likely to be predictable [49]. On the other hand, implementing change can provide opportunities for leaders to refine their leadership and management skills [50]. The interviews showed, however, that it in fact added to the managers’ worries over increased workload pressure when they already perceived themselves to be over-burdened with work. The interviews showed that managers needed support from senior managers and from the organizational structure in order to prioritize their work, but some organizations failed to provide such support. Several studies have identified that support from the organization is important for managers’ commitment to change [51, 52], that a lack of support can have negative consequences [53–55] and that lack of structure risks failure to prioritize limited resources in areas where improvements are most needed [35, 56]. Our results showed that the problem, as the managers perceived and expressed it, was to manage their day-to-day duties, which took a lot of time and consequently gave them less time to lead the change process. It seems that an overly managerial environment is a barrier to change processes [44], and our results indicated that the degree to which managers could handle the implementation process depended highly on to what extent there was resistance to change, reluctance to cooperate and lack of support and structure; at the same time, they had to keep up with the increasing pace of change in health care.

Most of the managers perceived themselves as skilled and competent when leading an implementation process. Some managers focused on the change process, that is, substituting one practice for another [50], rather than the implementation process, that is, the process of integrating a new practice within a particular setting [57]. However, the two concepts coexist, as implementation processes imply change [58, 59].

There was little support in the interviews for how new practices were transferred to work settings. The literature identifies four aspects of leadership as relevant for the implementation process [4, 60, 61]: The leader should establish clear goals and plans (first aspect), be knowledgeable about evidence-based intervention (second aspect), support staff efforts to learn to use evidence-based interventions and recognize their efforts in doing so (third aspect), and persist and move forward in the implementation process despite problems and challenges (fourth aspect) [4, 60, 61]. In our interviews, we found support for the first and second aspects of leadership but less support for the third and fourth aspects. Moreover, there was scarce support for the innovation construct in the i-PARIHS framework [3], *adapting evidence*, whereby explicit knowledge is blended with tacit, practice-based knowledge to suit a work setting [62]. One possible reason for this is that the field of disability lacks a tradition of implementing new practice in the work setting [37]. This may explain the managers’ experience of difficulties with this process. The managers claimed that they knew what to do when leading implementation processes but they were not explicit in describing how they did it. This may indicate a lack of the necessary knowledge and skills.

We found that managers of some habilitation organizations described the workplace culture as unique and very special. One possible explanation may be that patients in disability organizations have traditionally required a team of professionals from different medical fields to ensure that comprehensive care is provided [63]; furthermore, these patients have more complex health needs than the general population and therefore face greater difficulty in getting adequate health care [38, 64]. Ensuring comprehensive care is therefore challenging and complex for professionals supporting this population [38, 64].

Managers reported that the employees tended to automatically stick with old habits, instead of developing new, efficient practices in order to meet the patients’ individual needs. Empirical findings in various fields suggest that changing the context gives better results than changing unwanted behaviours [9, 65–69]. A systematic review by Kwasnicka et al. [66] explains behaviour change theories from initiation to maintenance and identifies five themes: *motives*, *self-regulation*, *resources*, *habits* and *contextual influences* [66]. In our study, managers identified habits and aspects of the workplace culture that appeared to influence employees’ automatic behaviour. This may indicate that the workplace culture encouraged the employees to engage in behaviours that did not meet the patients’ individual needs.

Previous evidence in the literature shows that the most important factors for optimizing implementation effectiveness across health care settings are organizational culture, networks and communication, leadership and resources [70]. Our study supports the significance of organizational culture and leadership and, moreover, it highlights the complex interplay between leadership and management and what consequences this has for the implementation process. In the literature, the debate is mainly about why and how leadership is similar to, or different from, management, and there are few empirical studies on how managers perform functions and their different roles in organizations [43–46]. Therefore, our study provides a useful contribution to the understanding of how managers strike a good balance between leadership and management to maximize their influence on the implementation process.

Limitations of the present study should be considered when interpreting the findings. One factor that may have affected the results is the selection of the participants. There is a risk that the managers who were not interested or had low motivation to change were the ones who declined to participate in the study. However, the managers participating in the study were open, with both positive and negative statements regarding the implementation process; this reduces the likelihood that managers with less motivation to change would have given views on the subject that had not already been expressed in the interviews reported here.

 The decision to perform telephone interviews could have positively influenced the number of respondents who agreed to participate in the study, since it made the arrangements more flexible. Telephone interviews have been shown to be a valid method for data collection [71]. Nonetheless, one could argue that the traditional face-to-face interview captures additional aspects of interaction. In our study, the benefit of flexibility with telephone interviews was considered to outweigh the risk of losing some aspects of interaction. All the authors helped to identify themes, which strengthens the internal validity of the results [72]. Other strengths were that the authors come from different occupational professions and have varied work experience. In addition, the first author transcribed all the interviews and became familiar with the material, which facilitated the analysis for all the researchers.

As the implementation process is a complex interplay between many factors, it is considered important not only to seek answers by studying the intervention itself but also to study factors such as leadership and organizational culture [14, 17]. A strength of this study is that the results shed light on this interplay.

## Conclusions

Our study found several important aspects of implementation processes and their impact from the experience of managers. They had to handle an imbalance between leading change and maintaining their managerial duties, which were interconnected with preconditions for change, while at the same time, keeping up with the fast pace in change. This resulted in ineffective implementation processes. Our study findings should empower managers to identify “what works” and to make strategies explicit for implementation processes. Overall, the study findings contribute to our understanding of these aspects of implementation processes in disability organizations and could inform other similar settings or organizations. Even though different contexts have different needs, the results of this study hold the potential to facilitate managers’ contributions in future implementation processes.

## Supplementary information

Additional file 1. Standards for reporting Qualitative Research (SRQR) checklist.pdf.

## Data Availability

The interviewed guide used during the current study are available from the corresponding author on request.

## Abbreviations

i-PARIHS: Integrated Promoting Action on Research Implementation in Health Services
SWAN: Structured Water Dance Intervention
TA: Reflexive Thematic Analysis
SRQR: The standards for reporting Qualitative research
