# Supplementary material for: Navigating change – managers’ experience of implementation processes in disability health care: a qualitative study
Source: BMC Health Serv Res. 2021 Jun 10;21:571. doi: 10.1186/s12913-021-06570-6 (PMC8190840; doi:10.1186/s12913-021-06570-6)
Supplement: Supplementary file 1 — Standards for reporting Qualitative Research (SRQR) checklist.pdf. [file 12913_2021_6570_MOESM1_ESM.pdf]

Standards for Reporting Qualitative Research: A Synthesis of Recommendations

Bridget C. O'Brien, PhD, Ilene B. Harris, PhD, Thomas J. Beckman, MD, Darcy A. Reed, MD, MPH, and David A. Cook, MD, MHPE

Abstract

Purpose

Standards for reporting exist for many types of quantitative research, but currently none exist for the broad spectrum of qualitative research. The purpose of the present study was to formulate and define standards for reporting qualitative research while preserving the requisite flexibility to accommodate various paradigms, approaches, and methods.

Method

The authors identified guidelines, reporting standards, and critical appraisal criteria for qualitative research by searching PubMed, Web of Science, and Google through July 2013; reviewing the reference lists of retrieved sources; and contacting experts. Specifically, two authors reviewed a sample of sources to generate an initial set of items that were potentially important in reporting qualitative research. Through an iterative process of reviewing sources, modifying the set of items, and coding all sources for items, the authors prepared a near-final list of items and descriptions and sent this list to five external reviewers for feedback. The final items and descriptions included in the reporting standards reflect this feedback.

Results

The Standards for Reporting Qualitative Research (SRQR) consists of 21 items. The authors define and explain key elements of each item and provide examples from recently published articles to illustrate ways in which the standards can be met.

Conclusions

The SRQR aims to improve the transparency of all aspects of qualitative research by providing clear standards for reporting qualitative research. These standards will assist authors during manuscript preparation, editors and reviewers in evaluating a manuscript for potential publication, and readers when critically appraising, applying, and synthesizing study findings.

Qualitative research contributes to the literature in many disciplines by describing, interpreting, and generating theories about social interactions and individual experiences as they occur in natural, rather than experimental, situations.[1–3] Some recent examples include studies of professional dilemmas,[4] medical students' early experiences of workplace learning,[5] patients' experiences of disease and interventions,[6–8] and patients' perspectives about incident disclosures.[9] The purpose of qualitative research is to understand the perspectives/experiences of individuals or groups and the contexts in which these perspectives or experiences are situated.[1,2,10]

Qualitative research is increasingly common and valued in the medical and medical education literature.[1,10–13] However, the quality of such research can be difficult to evaluate because of incomplete reporting of key elements.[14,15] *Quality* is multifaceted and includes consideration of the importance of the research question, the rigor of the research methods, the appropriateness and salience of the inferences, and the clarity and completeness of reporting.[16,17] Although there is much debate about standards for methodological rigor in qualitative research,[13,14,18–20] there is widespread agreement about the need for clear and complete reporting.[14,21,22] Optimal reporting would enable editors, reviewers, other researchers, and practitioners to critically appraise qualitative studies and apply and synthesize the results. One important step in improving the quality of reporting is to formulate and define clear reporting standards.

Authors have proposed guidelines for the quality of qualitative research, including those in the fields of medical education,[23–25] clinical and health services research,[26–28] and general education research.[29,30] Yet in nearly all cases, the authors do not describe how the guidelines were created, and often fail to distinguish reporting quality from the other facets of quality (e.g., the research question or methods). Several authors suggest standards for reporting qualitative research,[15,20,29–33] but their articles focus on a subset of qualitative data collection methods (e.g., interviews), fail to explain how the authors developed the reporting criteria, narrowly construe qualitative research (e.g., thematic analysis) in ways that may exclude other approaches, and/or lack specific examples to help others see how the standards might be achieved. Thus, there remains a compelling need for defensible and broadly applicable standards for reporting qualitative research.

We designed and carried out the present study to formulate and define standards for reporting qualitative research through a rigorous synthesis of published articles and expert recommendations.

Method

We formulated standards for reporting qualitative research by using a rigorous and systematic approach in which we reviewed previously proposed

Please see the end of this article for information about the authors.

Correspondence should be addressed to Dr. O'Brien, Office of Research and Development in Medical Education, UCSF School of Medicine, Box 3202, 1855 Folsom St., Suite 200, San Francisco, CA 94143-3202; e-mail: bridget.obrien@ucsf.edu.

Acad Med. 2014;89:00–00.
First published online
doi: 10.1097/ACM.0000000000000388

Supplemental digital content for this article is available at http://links.lww.com/ACADMED/A218.

Copyright © by the Association of American Medical Colleges. Unauthorized reproduction of this article is prohibited.

recommendations by experts in qualitative methods. Our research team consisted of two PhD researchers and one physician with formal training and experience in qualitative methods, and two physicians with experience, but no formal training, in qualitative methods.

We first identified previously proposed recommendations by searching PubMed, Web of Science, and Google using combinations of terms such as "qualitative methods," "qualitative research," "qualitative guidelines," "qualitative standards," and "critical appraisal" and by reviewing the reference lists of retrieved sources, reviewing the Equator Network,[22] and contacting experts. We conducted our first search in January 2007 and our last search in July 2013. Most recommendations were published in peer-reviewed journals, but some were available only on the Internet, and one was an interim draft from a national organization. We report the full set of the 40 sources reviewed in Supplemental Digital Appendix 1, found at http://links.lww.com/ACADMED/A218.

Two of us (B.O., I.H.) reviewed an initial sample of sources to generate a comprehensive list of items that were potentially important in reporting qualitative research (Draft A). All of us then worked in pairs to review all sources and code the presence or absence of each item in a given source. From Draft A, we then distilled a shorter list (Draft B) by identifying core concepts and combining related items, taking into account the number of times each item appeared in these sources. We then compared the items in Draft B with material in the original sources to check for missing concepts, modify accordingly, and add explanatory definitions to create a prefinal list of items (Draft C).

We circulated Draft C to five experienced qualitative researchers (see the acknowledgments) for review. We asked them to note any omitted or redundant items and to suggest improvements to the wording to enhance clarity and relevance across a broad spectrum of qualitative inquiry. In response to their reviews, we consolidated some items and made minor revisions to the wording of labels and definitions to create the final set of reporting standards—the Standards for Reporting Qualitative Research (SRQR)—summarized in Table 1.

To explicate how the final set of standards reflect the material in the original sources, two of us (B.O., D.A.C.) selected by consensus the 25 most complete sources of recommendations and identified which standards reflected the concepts found in each original source (see Table 2).

Results

The SRQR is a list of 21 items that we consider essential for complete, transparent reporting of qualitative research (see Table 1). As explained above, we developed these items through a rigorous synthesis of prior recommendations and concepts from published sources (see Table 2; see also Supplemental Digital Appendix 1, found at http://links.lww.com/ACADMED/A218) and expert review. These 21 items provide a framework and recommendations for reporting qualitative studies. Given the wide range of qualitative approaches and methodologies, we attempted to select items with broad relevance.

The SRQR includes the article's title and abstract (items 1 and 2); problem formulation and research question (items 3 and 4); research design and methods of data collection and analysis (items 5 through 15); results, interpretation, discussion, and integration (items 16 through 19); and other information (items 20 and 21). Supplemental Digital Appendix 2, found at http://links.lww.com/ACADMED/A218, contains a detailed explanation of each item, along with examples from recently published qualitative studies. Below, we briefly describe the standards, with a particular focus on those unique to qualitative research.

Titles, abstracts, and introductory material. Reporting standards for titles, abstracts, and introductory material (problem formulation, research question) in qualitative research are very similar to those for quantitative research, except that the results reported in the abstract are narrative rather than numerical, and authors rarely present a specific hypothesis.[29,30]

Research design and methods. Reporting on research design and methods of data collection and analysis highlights several distinctive features of qualitative research. Many of the criteria we reviewed focus not only on identifying and describing all aspects of the methods (e.g., approach, researcher characteristics and role, sampling strategy, context, data collection and analysis) but also on justifying each choice.[13,14] This ensures that authors make their assumptions and decisions transparent to readers. This standard is less commonly expected in quantitative research, perhaps because most quantitative researchers share positivist assumptions and generally agree about standards for rigor of various study designs and sampling techniques.[14] Just as quantitative reporting standards encourage authors to describe how they implemented methods such as randomization and measurement validity, several qualitative reporting criteria recommend that authors describe how they implemented a presumably familiar technique in their study rather than simply mentioning the technique.[10,14,32] For example, authors often state that data collection occurred until saturation, with no mention of how they defined and recognized saturation. Similarly, authors often mention an "iterative process," with minimal description of the nature of the iterations. The SRQR emphasizes the importance of explaining and elaborating on these important processes. Nearly all of the original sources recommended describing the characteristics and role of the researcher (i.e., reflexivity). Members of the research team often form relationships with participants, and analytic processes are highly interpretive in most qualitative research. Therefore, reviewers and readers must understand how these relationships and the researchers' perspectives and assumptions influenced data collection and interpretation.[15,23,26,34]

Results. Reporting of qualitative research results should identify the main analytic findings. Often, these findings involve interpretation and contextualization, which represent a departure from the tradition in quantitative studies of objectively reporting results. The presentation of results often varies with the specific qualitative approach and methodology; thus, rigid rules for reporting qualitative findings are inappropriate. However, authors

Copyright © by the Association of American Medical Colleges. Unauthorized reproduction of this article is prohibited.

Table 1

Standards for Reporting Qualitative Research (SRQR)[a]

No.	Topic	Item
	**Title and abstract**	
S1	Title	Concise description of the nature and topic of the study Identifying the study as qualitative or indicating the approach (e.g., ethnography, grounded theory) or data collection methods (e.g., interview, focus group) is recommended
S2	Abstract	Summary of key elements of the study using the abstract format of the intended publication; typically includes background, purpose, methods, results, and conclusions
	**Introduction**	
S3	Problem formulation	Description and significance of the problem/phenomenon studied; review of relevant theory and empirical work; problem statement
S4	Purpose or research question	Purpose of the study and specific objectives or questions
	**Methods**	
S5	Qualitative approach and research paradigm	Qualitative approach (e.g., ethnography, grounded theory, case study, phenomenology, narrative research) and guiding theory if appropriate; identifying the research paradigm (e.g., postpositivist, constructivist/ interpretivist) is also recommended; rationale[b]
S6	Researcher characteristics and reflexivity	Researchers' characteristics that may influence the research, including personal attributes, qualifications/experience, relationship with participants, assumptions, and/or presuppositions; potential or actual interaction between researchers' characteristics and the research questions, approach, methods, results, and/or transferability
S7	Context	Setting/site and salient contextual factors; rationale[b]
S8	Sampling strategy	How and why research participants, documents, or events were selected; criteria for deciding when no further sampling was necessary (e.g., sampling saturation); rationale[b]
S9	Ethical issues pertaining to human subjects	Documentation of approval by an appropriate ethics review board and participant consent, or explanation for lack thereof; other confidentiality and data security issues
S10	Data collection methods	Types of data collected; details of data collection procedures including (as appropriate) start and stop dates of data collection and analysis, iterative process, triangulation of sources/methods, and modification of procedures in response to evolving study findings; rationale[b]
S11	Data collection instruments and technologies	Description of instruments (e.g., interview guides, questionnaires) and devices (e.g., audio recorders) used for data collection; if/how the instrument(s) changed over the course of the study
S12	Units of study	Number and relevant characteristics of participants, documents, or events included in the study; level of participation (could be reported in results)
S13	Data processing	Methods for processing data prior to and during analysis, including transcription, data entry, data management and security, verification of data integrity, data coding, and anonymization/deidentification of excerpts
S14	Data analysis	Process by which inferences, themes, etc., were identified and developed, including the researchers involved in data analysis; usually references a specific paradigm or approach; rationale [b]
S15	Techniques to enhance trustworthiness	Techniques to enhance trustworthiness and credibility of data analysis (e.g., member checking, audit trail, triangulation); rationale[b]
	**Results/findings**	
S16	Synthesis and interpretation	Main findings (e.g., interpretations, inferences, and themes); might include development of a theory or model, or integration with prior research or theory
S17	Links to empirical data	Evidence (e.g., quotes, field notes, text excerpts, photographs) to substantiate analytic findings
	**Discussion**	
S18	Integration with prior work, implications, transferability, and contribution(s) to the field	Short summary of main findings; explanation of how findings and conclusions connect to, support, elaborate on, or challenge conclusions of earlier scholarship; discussion of scope of application/ generalizability; identification of unique contribution(s) to scholarship in a discipline or field
S19	Limitations	Trustworthiness and limitations of findings

(*Table continues*)

Copyright © by the Association of American Medical Colleges. Unauthorized reproduction of this article is prohibited.

Table 1
(Continued)

No.	Topic	Item
	**Other**	
S20	Conflicts of interest	Potential sources of influence or perceived influence on study conduct and conclusions; how these were managed
S21	Funding	Sources of funding and other support; role of funders in data collection, interpretation, and reporting

[a]The authors created the SRQR by searching the literature to identify guidelines, reporting standards, and critical appraisal criteria for qualitative research; reviewing the reference lists of retrieved sources; and contacting experts to gain feedback. The SRQR aims to improve the transparency of all aspects of qualitative research by providing clear standards for reporting qualitative research.
[b]The rationale should briefly discuss the justification for choosing that theory, approach, method, or technique rather than other options available, the assumptions and limitations implicit in those choices, and how those choices influence study conclusions and transferability. As appropriate, the rationale for several items might be discussed together.

should provide evidence (e.g., examples, quotes, or text excerpts) to substantiate the main analytic findings.[20,29]

Discussion. The discussion of qualitative results will generally include connections to existing literature and/or theoretical or conceptual frameworks, the scope and boundaries of the results (transferability), and study limitations.[10–12,28] In some qualitative traditions, the results and discussion may not have distinct boundaries; we recommend that authors include the substance of each item regardless of the section in which it appears.

Discussion

The purpose of the SRQR is to improve the quality of reporting of qualitative research studies. We hope that these 21 recommended reporting standards will assist authors during manuscript preparation, editors and reviewers in evaluating a manuscript for potential publication, and readers when critically appraising, applying, and synthesizing study findings. As with other reporting guidelines,[35–37] we anticipate that the SRQR will evolve as it is applied and evaluated in practice. We welcome suggestions for refinement.

Qualitative studies explore "how?" and "why?" questions related to social or human problems or phenomena.[10,38] Purposes of qualitative studies include understanding meaning from participants' perspectives (How do they interpret or make sense of an event, situation, or action?); understanding the nature and influence of the context surrounding events or actions; generating theories about new or poorly understood events, situations, or actions; and understanding the processes that led to a desired (or undesired) outcome.[38] Many different approaches (e.g., ethnography, phenomenology, discourse analysis, case study, grounded theory) and methodologies (e.g., interviews, focus groups, observation, analysis of documents) may be used in qualitative research, each with its own assumptions and traditions.[1,2] A strength of many qualitative approaches and methodologies is the opportunity for flexibility and adaptability throughout the data collection and analysis process. We endeavored to maintain that flexibility by intentionally defining items to avoid favoring one approach or method over others. As such, we trust that the SRQR will support all approaches and methods of qualitative research by making reports more explicit and transparent, while still allowing investigators the flexibility to use the study design and reporting format most appropriate to their study. It may be helpful, in the future, to develop approach-specific extensions of the SRQR, as has been done for guidelines in quantitative research (e.g., the CONSORT extensions).[37]

Limitations, strengths, and boundaries

We deliberately avoided recommendations that define methodological rigor, and therefore it would be inappropriate to use the SRQR to judge the quality of research methods and findings. Many of the original sources from which we derived the SRQR were intended as criteria for methodological rigor or critical appraisal rather than reporting; for these, we inferred the information that would be needed to evaluate the criterion. Occasionally, we found conflicting recommendations in the literature (e.g., recommending specific techniques such as multiple coders or member checking to demonstrate trustworthiness); we resolved these conflicting recommendations through selection of the most frequent recommendations and by consensus among ourselves.

Some qualitative researchers have described the limitations of checklists as a means to improve methodological rigor.[13] We nonetheless believe that a checklist for reporting standards will help to enhance the transparency of qualitative research studies and thereby advance the field.[29,39]

Strengths of this work include the grounding in previously published criteria, the diversity of experience and perspectives among us, and critical review by experts in three countries.

Implications and application

Similar to other reporting guidelines,[35–37] the SRQR may be viewed as a starting point for defining reporting standards in qualitative research. Although our personal experience lies in health professions education, the SRQR is based on sources originating in diverse health care and non-health-care fields. We intentionally crafted the SRQR to include various paradigms, approaches, and methodologies used in qualitative research. The elaborations offered in

Copyright © by the Association of American Medical Colleges. Unauthorized reproduction of this article is prohibited.

Table 2

Alignment of the 21 Standards for Reporting Qualitative Research (SRQR) With Recommendations From 25 Original Sources[a]

		Reference no.[b]																							
No.	Topic	11,12	15[c]	19	20[c]	23	24,25[d]	26	27	29[c,d]	30[c,d]	31[c]	32[c]	33	34	41	42	43	44[c]	45	46	47	48	49	50
S1	Title						*	*		*														*	
S2	Abstract						*			*	*			*											
S3	Problem formulation				*	*	*	*	*	*	*	*		*	*	*	*	*			*			*	*
S4	Purpose or research question	*	*	*	*	*	*	*	*	*	*	*	*	*	*	*	*	*		*	*	*	*	*	*
S5	Qualitative approach and research paradigm	*	*	*	*	*	*	*		*	*		*	*		*	*	*			*	*	*	*	*
S6	Researcher characteristics, reflexivity	*	*	*	*	*	*	*	*	*		*	*	*		*	*	*	*	*	*	*	*	*	*
S7	Context		*	*	*	*	*	*	*	*	*	*		*		*	*		*	*	*		*		*
S8	Sampling strategy	*	*	*	*	*	*	*	*	*	*	*	*	*	*	*	*	*	*	*	*		*	*	
S9	Ethical issues pertaining to human subjects	*			*		*			*	*		*	*		*	*	*		*	*	*			*
S10	Data collection methods	*	*	*	*	*	*	*	*	*	*	*	*	*	*	*	*	*	*	*	*	*	*	*	*
S11	Data collection instruments/ technologies	*	*				*			*	*	*	*	*		*		*			*				
S12	Units of study	*	*		*		*	*		*	*	*	*	*		*					*			*	
S13	Data processing	*				*	*	*		*	*	*	*				*				*				*
S14	Data analysis	*	*	*	*	*	*	*	*	*	*	*	*	*	*	*	*	*	*	*	*	*	*	*	*
S15	Techniques to enhance trustworthiness	*	*	*	*	*	*	*	*	*	*	*	*	*	*	*	*	*	*	*	*		*	*	*
S16	Synthesis and interpretation	*	*		*	*	*	*	*	*	*	*	*	*		*	*	*			*	*	*	*	*
S17	Links to empirical data	*	*		*	*	*	*	*	*	*	*		*		*	*	*	*			*	*		*
S18	Integration with prior work, implications, transferability, and contribution(s)	*		*	*	*	*	*	*	*	*	*	*	*		*	*	*	*	*	*		*	*	*
S19	Limitations	*			*	*	*	*		*				*		*	*	*			*				*
S20	Conflicts of interest						*			*															
S21	Funding									*						*								*	

[a]The authors created the SRQR by searching the literature to identify guidelines, reporting standards, and critical appraisal criteria for qualitative research; reviewing the reference lists of retrieved sources; and contacting experts to gain feedback. The SRQR aims to improve the transparency of all aspects of qualitative research by providing clear standards for reporting qualitative research. In the table, the asterisks indicate which sources mentioned which topics.
[b]The numbers in column headings are the numbers of the citations in the reference list at the end of this report. Those citations are of original sources describing criteria for reporting and/or critical appraisal of qualitative research, which the authors used in creating the SRQR.
[c]Focuses on reporting standards (all other sources focus on quality standards or guidelines for critical review/evaluation).
[d]Addresses quantitative and qualitative research.

Supplemental Digital Appendix 2 (see http://links.lww.com/ACADMED/A218) should provide sufficient description and examples to enable both novice and experienced researchers to use these standards. Thus, the SRQR should apply broadly across disciplines, methodologies, topics, study participants, and users.

Copyright © by the Association of American Medical Colleges. Unauthorized reproduction of this article is prohibited.

The SRQR items reflect information essential for inclusion in a qualitative research report, but should not be viewed as prescribing a rigid format or standardized content. Individual study needs, author preferences, and journal requirements may necessitate a different sequence or organization than that shown in Table 1. Journal word restrictions may prevent a full exposition of each item, and the relative importance of a given item will vary by study. Thus, although all 21 standards would ideally be reflected in any given report, authors should prioritize attention to those items that are most relevant to the given study, findings, context, and readership.

Application of the SRQR need not be limited to the writing phase of a given study. These standards can assist researchers in planning qualitative studies and in the careful documentation of processes and decisions made throughout the study. By considering these recommendations early on, researchers may be more likely to identify the paradigm and approach most appropriate to their research, consider and use strategies for ensuring trustworthiness, and keep track of procedures and decisions.

Journal editors can facilitate the review process by providing the SRQR to reviewers and applying its standards, thus establishing more explicit expectations for qualitative studies. Although the recommendations do not address or advocate specific approaches, methods, or quality standards, they do help reviewers identify information that is missing from manuscripts.

As authors and editors apply the SRQR, readers will have more complete information about a given study, thus facilitating judgments about the trustworthiness, relevance, and transferability of findings to their own context and/or to related literature. Complete reporting will also facilitate meaningful synthesis of qualitative results across studies.[40] We anticipate that such transparency will, over time, help to identify previously unappreciated gaps in the rigor and relevance of research findings. Investigators, editors, and educators can then work to remedy these deficiencies and, thereby, enhance the overall quality of qualitative research.

Acknowledgments: The authors thank Margaret Bearman, PhD, Calvin Chou, MD, PhD, Karen Hauer, MD, Ayelet Kuper, MD, DPhil, Arianne Teherani, PhD, and participants in the UCSF weekly educational scholarship works-in-progress group (ESCape) for critically reviewing the Standards for Reporting Qualitative Research.

Funding/Support: This study was funded in part by a research review grant from the Society for Directors of Research in Medical Education.

Other disclosures: None reported.

Ethical approval: Reported as not applicable.

Disclaimer: The funding agency had no role in the study design, analysis, interpretation, writing of the manuscript, or decision to submit the manuscript for publication.

Dr. O'Brien is assistant professor, Department of Medicine and Office of Research and Development in Medical Education, University of California, San Francisco, School of Medicine, San Francisco, California.

Dr. Harris is professor and head, Department of Medical Education, University of Illinois at Chicago College of Medicine, Chicago, Illinois.

Dr. Beckman is professor of medicine and medical education, Department of Medicine, Mayo Clinic College of Medicine, Rochester, Minnesota.

Dr. Reed is associate professor of medicine and medical education, Department of Medicine, Mayo Clinic College of Medicine, Rochester, Minnesota.

Dr. Cook is associate director, Mayo Clinic Online Learning, research chair, Mayo Multidisciplinary Simulation Center, and professor of medicine and medical education, Mayo Clinic College of Medicine, Rochester, Minnesota.

References

1 Lingard L, Kennedy TJ. Qualitative research in medical education. In: Swanwick T, ed. Understanding Medical Education: Evidence, Theory and Practice. Oxford, UK: Wiley-Blackwell; 2010:323–335.
2 Harris IB. Qualitative methods. In: Norman GR, van der Vleuten CPM, Newble DI, eds. International Handbook of Research in Medical Education. Dordrecht, Netherlands: Kluwer Academic Publishers; 2002:45–95.
3 Denzin N, Lincoln Y. Introduction: The discipline and practice of qualitative research. In: The Sage Handbook of Qualitative Research. 3rd ed. Thousand Oaks, Calif: Sage Publications, Inc.; 2005:1–32.
4 Ginsburg S, Bernabeo E, Ross KM, Holmboe ES. "It depends": Results of a qualitative study investigating how practicing internists approach professional dilemmas. Acad Med. 2012;87:1685–1693.
5 Yardley S, Brosnan C, Richardson J, Hays R. Authentic early experience in medical education: A socio-cultural analysis identifying important variables in learning interactions within workplaces. Adv Health Sci Educ Theory Pract. 2013;18:873–891.
6 Embuldeniya G, Veinot P, Bell E, et al. The experience and impact of chronic disease peer support interventions: A qualitative synthesis. Patient Educ Couns. 2013;92:3–12.
7 Pinnock H, Kendall M, Murray SA, et al. Living and dying with severe chronic obstructive pulmonary disease: Multi-perspective longitudinal qualitative study. BMJ. 2011;342:d142.
8 Brady MC, Clark AM, Dickson S, Paton G, Barbour RS. Dysarthria following stroke: The patient's perspective on management and rehabilitation. Clin Rehabil. 2011;25:935–952.
9 Iedema R, Allen S, Britton K, et al. Patients' and family members' views on how clinicians enact and how they should enact incident disclosure: The "100 patient stories" qualitative study. BMJ. 2011;343:d4423.
10 Kuper A, Reeves S, Levinson W. An introduction to reading and appraising qualitative research. BMJ. 2008;337:404–407.
11 Giacomini M, Cook, DJ. Users' guides to the medical literature: XXIII. Qualitative research in health care A. Are the results of the study valid? JAMA. 2000;284:357–362.
12 Giacomini M, Cook, DJ. Users' guides to the medical literature: XXIII. Qualitative research in health care B. What are the results and how do they help me care for my patients? JAMA. 2000;284:478–482.
13 Barbour RS. Checklists for improving rigour in qualitative research: A case of the tail wagging the dog? BMJ. 2001;322:1115–1117.
14 Dunt D, McKenzie R. Improving the quality of qualitative studies: Do reporting guidelines have a place? Fam Pract. 2012;29:367–369.
15 Tong A, Sainsbury P, Craig J. Consolidated criteria for reporting qualitative research (COREQ): A 32-item checklist for interviews and focus groups. Int J Qual Health Care. 2007;19:349–357.
16 Cook DA, Bowen JL, Gerrity MS, et al. Proposed standards for medical education submissions to the Journal of General Internal Medicine. J Gen Intern Med. 2008;23:908–913.
17 Tracy SJ. Qualitative quality: Eight "big-tent" criteria for excellent qualitative research. Qual Inq. 2010;16:837–851.
18 Lincoln YS. Emerging criteria for quality in qualitative and interpretive research. Qual Inq. 1995;1:275–289.
19 Mays N, Pope C. Qualitative research in health care. Assessing quality in qualitative research. BMJ. 2000;320:50–52.
20 Burns N. Standards for qualitative research. Nurs Sci Q. 1989;2:44–52.
21 Ryan GW. What Are Standards of Rigor for Qualitative Research? 2005. http://www.wjh.harvard.edu/nsfqual/Ryan%20Paper.pdf. Accessed April 20, 2014.
22 The EQUATOR Network: Enhancing the quality and transparency of health research. http://www.equator-network.org. Accessed April 6, 2014.
23 Côté L, Turgeon J. Appraising qualitative research articles in medicine and medical education. Med Teach. 2005;27:71–75.
24 Bordage G, Caelleigh AS. A tool for reviewers: "Review criteria for research manuscripts." Acad Med. 2001;76:904–951.
25 Task Force of Academic Medicine and the GEA-RIME Committee. Appendix 1: Checklist of review criteria. Acad Med. 2001;76:958–959.
26 Malterud K. Qualitative research: Standards, challenges, and guidelines. Lancet. 2001;358:483–488.

Copyright © by the Association of American Medical Colleges. Unauthorized reproduction of this article is prohibited.

27 Inui TS, Frankel RM. Evaluating the quality of qualitative research: A proposal pro tem. J Gen Intern Med. 1991;6:485–486.

28 Devers KJ. How will we know" good" qualitative research when we see it? Beginning the dialogue in health services research. Health Serv Res. 1999;34:1153.

29 Duran RP, Eisenhart MA, Erickson FD, et al. Standards for reporting on empirical social science research in AERA publications. Educ Res. 2006;35:33–40.

30 Newman M, Elbourne D. Improving the usability of educational research: Guidelines for the reporting of primary empirical research studies in education (The REPOSE Guidelines). Eval Res Educ. 2004;18:201–212.

31 Knafl KA, Howard MJ. Interpreting and reporting qualitative research. Res Nurs Health. 1984;7:17–24.

32 Kitto SC, Chesters J, Grbich C. Quality in qualitative research. Med J Aust. 2008;188:243–246.

33 Rowan M, Huston P. Qualitative research articles: Information for authors and peer reviewers. CMAJ. 1997;157:1442–1446.

34 Cohen D, Crabtree B. Guidelines for designing, analyzing, and reporting qualitative research. Qualitative Research Guidelines Project, Robert Wood Johnson Foundation. 2006. http://qualres.org/HomeGuid-3868.html. Accessed April 6, 2014.

35 Elm E von, Altman DG, Egger M, Pocock SJ, Gøtzsche PC, Vandenbroucke JP. Strengthening the reporting of observational studies in epidemiology (STROBE) statement: Guidelines for reporting observational studies. BMJ. 2007;335:806–808.

36 Davidoff F, Batalden P, Stevens D, Ogrinc G, Mooney S; SQUIRE Development Group. Publication guidelines for quality improvement in health care: Evolution of the SQUIRE project. Qual Saf Health Care. 2008;17(suppl 1):i3–i9.

37 Schulz KF, Altman DG, Moher D; CONSORT Group. CONSORT 2010 statement: Updated guidelines for reporting parallel group randomised trials. BMJ. 2010;340:c332.

38 Maxwell JA. Designing a qualitative study. In: Bickman L, Bog D, eds. The SAGE Handbook of Applied Social Research Methods. 2nd ed. Sage Publications, Inc.; 2009:214–253.

39 Meyrick J. What is good qualitative research? A first step towards a comprehensive approach to judging rigour/quality. J Health Psychol. 2006;11:799–808.

40 Bearman M, Dawson P. Qualitative synthesis and systematic review in health professions education. Med Educ. 2013;47:252–260.

References Cited Only in Table 2

41 Attree P, Milton B. Critically appraising qualitative research for systematic reviews. Evid Policy. 2006;2:109–126.

42 Blaxter M. Criteria for the evaluation of qualitative research. Med Sociol News. 1996;22:34–37.

43 Critical Appraisal Skills Programme (CASP). Qualitative Research Checklist. 2013. http://www.casp-uk.net/wp-content/uploads/2011/11/casp-qualitative-research-checklist-31.05.13.pdf#!casp-tools-checklists/c18f8. Accessed April 6, 2014.

44 Frambach JM, van der Vleuten CP, Durning SJ. AM last page. Quality criteria in qualitative and quantitative research. Acad Med. 2013;88:552.

45 Kuper A, Lingard L, Levinson W. Critically appraising qualitative research. BMJ. 2008;337:687–689.

46 Law M, Stewart D, Letts L, Pollock N, Bosch J, Westmorland M. Guidelines for the critical review of qualitative studies. McMaster University Occupational Therapy Evidence-Based Practice Research Group. 1998. http://www.usc.edu/hsc/ebnet/res/Guidelines.pdf. Accessed April 20, 2014.

47 Pearson A, Field J, Jordan Z. Appendix 2: Critical appraisal tools. In: Evidence-Based Clinical Practice in Nursing and Health Care: Assimilating Research, Experience and Expertise. Oxford, UK: Blackwell Publishing Ltd.; 2009:177–182. http://onlinelibrary.wiley.com/doi/10.1002/9781444316544.app2/summary. Accessed April 13, 2014.

48 Popay J, Rogers A, Williams G. Rationale and standards for the systematic review of qualitative literature in health services research. Qual Health Res. 1998;8:341–351.

49 Sandelowski M, Barroso J. Writing the proposal for a qualitative research methodology project. Qual Health Res. 2003;13:781–820.

50 Stige B, Malterud K, Midtgarden T. Toward an agenda for evaluation of qualitative research. Qual Health Res. 2009;19:1504–1516.

Copyright © by the Association of American Medical Colleges. Unauthorized reproduction of this article is prohibited.
